# Exploring Habits in Anorexia Nervosa: Promise, Pitfalls, and Progress

**DOI:** 10.1007/s11920-025-01588-7

**Published:** 2025-02-28

**Authors:** Karin Foerde

**Affiliations:** Department of Psychology, Brain and Cognition, University of Amsterdam, Nieuwe Achtergracht 129, Amsterdam, 1001 NK The Netherlands

**Keywords:** Anorexia nervosa, Habits, Goal-directed behavior, Task-based assessment, Maladaptive behaviors, Frontostriatal circuits

## Abstract

**Purpose of Review:**

Habits, characterized by automaticity and insensitivity to outcomes, may be key to the persistence of maladaptive behaviors in anorexia nervosa (AN). This review examines the status of habit research in AN, focusing on insights from task-based assessments.

**Recent Findings:**

Findings indicate dysfunction in the frontostriatal circuits associated with habitual and goal-directed behaviors, with some studies linking neural disturbances to habit measures or clinically relevant behaviors. Heightened habitual tendencies in AN have consistently been reported using self-reports, while research utilizing experimental paradigms has yielded mixed results and efforts to capture real-world habits in AN remain limited.

**Summary:**

Some experimental paradigms appear more sensitive than others, but all face challenges associated with studying habits in the lab. Promising new approaches will need to be adopted and efforts made to capture real-world habits. Understanding which habits are problematic, when in illness and for whom they dominate, could make good on the promise of habit-focused treatments for AN.

## Introduction

Anorexia nervosa (AN) is a severe psychiatric illness with a high rate of mortality [1]. Long-term outcomes are often disappointing, marked by high rates of relapse and chronicity [2, 3, 4]. Understanding why maladaptive behaviors persist, even in individuals seeking recovery, is crucial. To do so researchers have turned to the neurocognitive mechanisms underlying these behaviors, increasingly with particular attention to habits. This review examines the status of neurocognitive research on the role of habits in AN, focusing on recent insights from task-based assessments and their contributions to advancing our understanding. Recent studies of the relevant neural circuitry and general methods of assessing habits will be briefly reviewed, followed by a more detailed analysis of the main task-based approaches. Finally, the challenges to this program of research and approaches for meeting those challenges will be outlined.

### The Habit Framework as a Guide for Studying Neurocognitive Mechanisms of Anorexia Nervosa

**Fig. 1:**
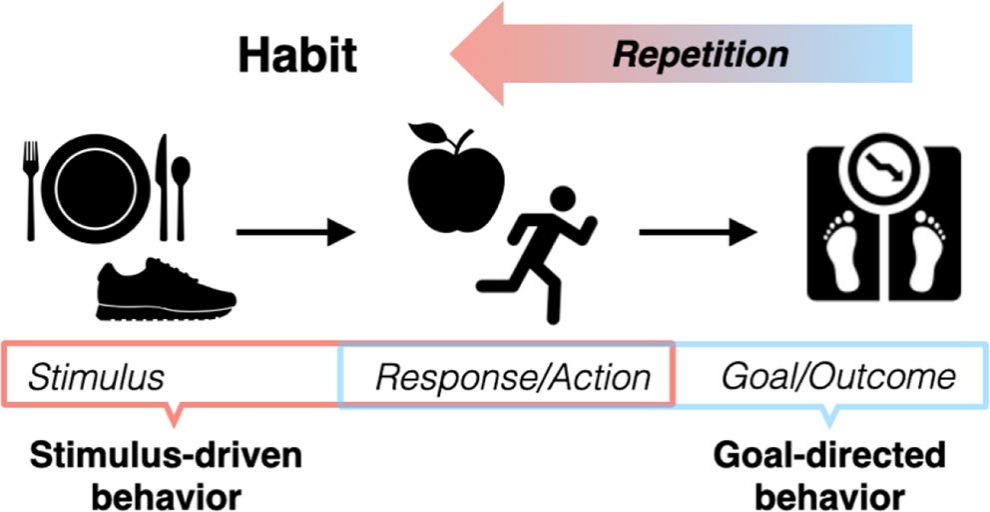
Habit development. Habits are learned behaviors that emerge through repetition. Behavior may begin as an intentional and goal-directed action (dieting to lose weight) in response to a stimulus or setting (food/mealtime) and reinforced by a rewarding outcome (weight loss). As the behavior is repeated and reinforced, it may shift to a more automated response triggered by food stimuli or contexts, regardless of whether the outcome of the action is still desired

Habits are learned behaviors that through repetition become fixed and relatively insensitive to their outcomes [5, 6]. This process is adaptive, allowing efficient execution of frequent behaviors, freeing cognitive resources for other tasks. However, in psychiatric illness, this learning process may be co-opted towards maladaptive ends. Habits may begin as intentional, goal-directed behaviors, guided by response-outcome associations but, through repeated reinforcement, shift to automatic responses triggered by specific cues or contexts (i.e., guided by stimulus-response associations) [6, 7, 8, 9, 10] (see Fig. 1).

Maladaptive eating behaviors observed in AN, such as extreme food restriction, align with this habit framework. Restrictive eating behavior may begin as casual dieting, reinforced by positive feedback or a sense of achievement, but over time it may persist without reinforcement—despite awareness of its harmful consequences [11, 12]. Similar trajectories may apply to other behaviors, such as excessive exercise [13]. The automaticity of habits is also relevant. Behavioral routines can become “chunked” into sequences that, once initiated, continue until conclusion without deliberate control [14]. In a similar vein, eating behavior in AN can take on a relatively stereotyped character, including limited diet variety [15, 16, 17] and rigid routines around eating [18, 19]. The challenge associated with avoiding automatic behavior patterns and the effort required to modify or eliminate entrenched routines, is a challenge familiar even in everyday habits (e.g., when you take the usual route home instead of buying groceries as intended).

**Fig. 2:**
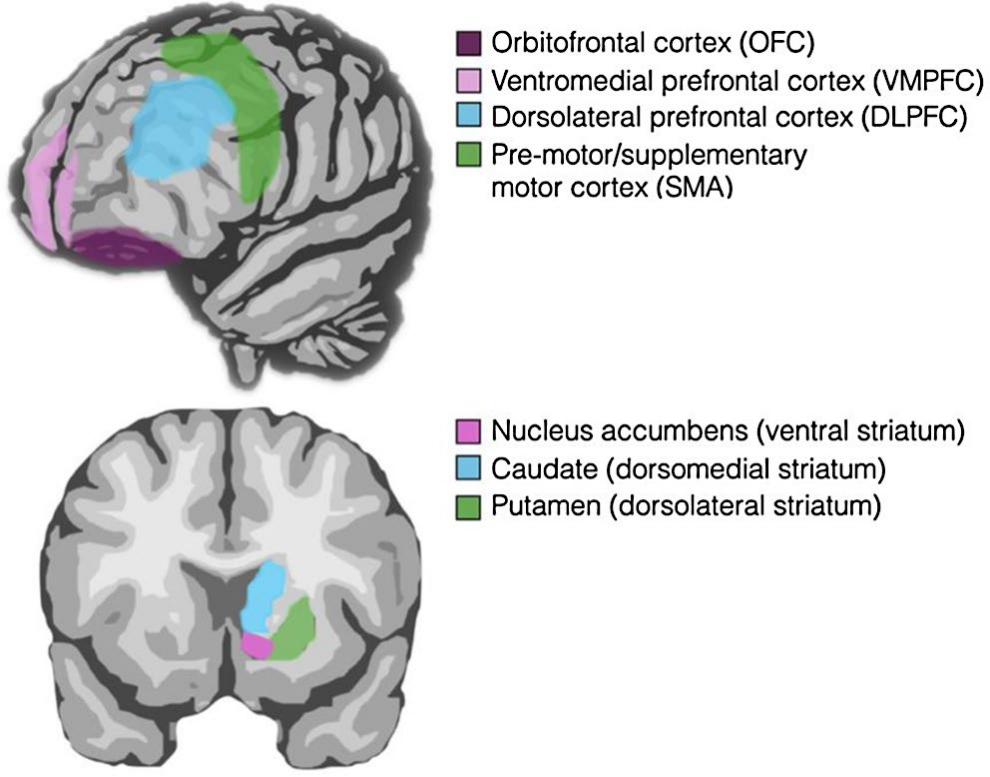
Frontostriatal circuits loops involved in habitual and goal-directed behavior

Considering behaviors as habits is only a starting point for understanding the neurocognitive mechanisms of AN and improving treatment [12, 20, 21, 22]. The habit model provides a framework for investigating AN at neural and behavioral levels, leveraging extensive research on habits in psychology and neuroscience. The distinction between goal-directed and habitual behaviors has been studied in the context of learning theory, cognitive neuroscience, and computational models [6, 8, 9, 10, 23, 24, 25, 26, 27, 28]. Behavioral paradigms to measure habitual and goal-directed behavior [29] complement self-report measures [30], while studies across animals and humans [27, 31, 32, 33, 34, 35] have identified ventral and dorsal frontostriatal circuits (Fig. 2) [6, 36, 37] and dopamine as critical for learning and behavior [38, 39, 40, 41].

Building on this literature, research on AN has begun to examine the neural circuits involved in habit formation and goal-directed behavior. Recent studies have linked neural abnormalities to habitual tendencies, and a small, but growing, number of behavioral experiments directly examine habits in this population. These efforts will be the focus of the current review.

### Abnormalities in Frontostriatal Circuits Associated with Habitual and Goal-Directed Behavior

If dysregulation of habitual vs. goal-directed control of behavior plays a role in AN, neural circuits supporting these behaviors should show evidence of dysfunction. Indeed, structural neuroimaging studies, across a variety of measures [42, 43, 44, 45, 46, 47, 48], have identified abnormalities in frontostriatal circuits. Functional neuroimaging studies, both resting state [21, 49, 50, 51] and task-based [52, 53, 54, 55, 56, 57, 58, 59, 60, 61], provide additional evidence for abnormalities in these circuits.

Most studies have not, thus far, assessed habits directly, but some recent studies have attempted to do so indirectly. One study reported greater white-matter connectivity measures in habit-associated pre-motor/SMA-putamen circuits among individuals with AN, relative to healthy controls, and that connectivity measures were associated with severity on the Rituals subscale of the Yale-Brown-Cornell Eating Disorder Scale [46] among individuals with AN. Another study reported an association between cerebral metabolite measures in inferior frontal cortex and Automaticity scores on the Creature of Habit Scale^1^ among individuals with the binge-eating/purging subtype of AN [62]. Finally, Lloyd et al. [63] reported (in exploratory analyses) that among individuals with AN, choice-related activity on a Food Choice Task [64] in the anterior caudate region of the striatum was associated with scores on the Self-Report Habit Index (SRHI). Other recent neuroimaging studies have linked abnormalities in frontostriatal circuits to weight or food intake. For example, Gorrell et al. [65] found that OFC response among patients with AN when expecting a sweet taste was related to longer-term BMI and BMI change after treatment. A set of studies using the Food Choice task showed associations between food choice-related activation in the caudate and caloric intake in a laboratory meal [56] and between pre- to post-treatment changes in caudate activity and changes in food choices [55] among individuals with AN. Although these studies do not affirm a role for habits in food restriction or other maladaptive behaviors, they demonstrate that dysfunction in brain circuits implicated in habitual and goal-directed behaviors is related to self-reported habitual tendencies or illness.

Notably, frontostriatal circuits are critically innervated by dopamine [66], which is thought to be important for both habitual and goal-directed behaviors [39, 40, 67]. Dopamine plays a role in propagating learning signals and conveying motivational significance [41, 68, 69, 70, 71] and has been linked to feeding in animals [72, 73]. Consequently, it is speculated that dopaminergic dysfunction plays some role in AN and, though they do not measure dopamine function, fMRI studies are often discussed as implicating a role for dopamine. Few studies have directly measured dopamine function in individuals with AN and have yielded mixed result—increased, decreased, or no differences in dopamine levels or activity [74, 75, 76]. Until recently, examination of dopamine has depended on somewhat invasive measures (lumbar puncture, PET, blood draws), but developments in imaging techniques [77] will likely yield more investigations of dopamine function in AN (e.g [78]).

### Studying Habits in Anorexia Nervosa: Self-Report

Several studies testing the role of habits in AN have used the Self-Reported Habit Index (SRHI; [30] and found that habit strength of food restriction predicted (self-reported) restriction [79], that interventions to interrupt maladaptive eating routines by interfering with trigger cues can decrease habit strength [22], and that longer illness duration and illness severity is associated with greater habit strength [80]. These studies established that behaviors central to illness were characterized as habitual in nature by individuals with AN.

A recent study used ecological momentary assessments (EMA) to assess the frequency of both food and non-food related (hygiene) habits in daily life [81] and found that patients with AN reported more food *and* non-food habits relative to healthy controls. However, surprisingly, patients with AN did not report higher habit strength, per the SRHI, for those habits relative to controls, nor for food relative to non-food habits. Perhaps the sample, comprising mainly adolescents with a short duration of illness, or the types of habits it is possible to report in an inpatient setting, played a role. Regardless, capturing habits more directly, as they unfold in the real world, will be important to better understand how they contribute to illness.

### Studying Habits in Anorexia Nervosa: Learning Tasks

A promise of the habit framework is that it might illuminate the mechanisms of maladaptive eating (and other) behaviors—how they arise, are maintained, why they are so persistent, and why they are prone to reoccurrence after treatment. Self-report questionnaires are not informative on this topic and more experimental behavioral research to examine habit mechanisms in AN has been encouraged [21]. Yet studies remain scarce and results mixed.

**Fig. 3:**
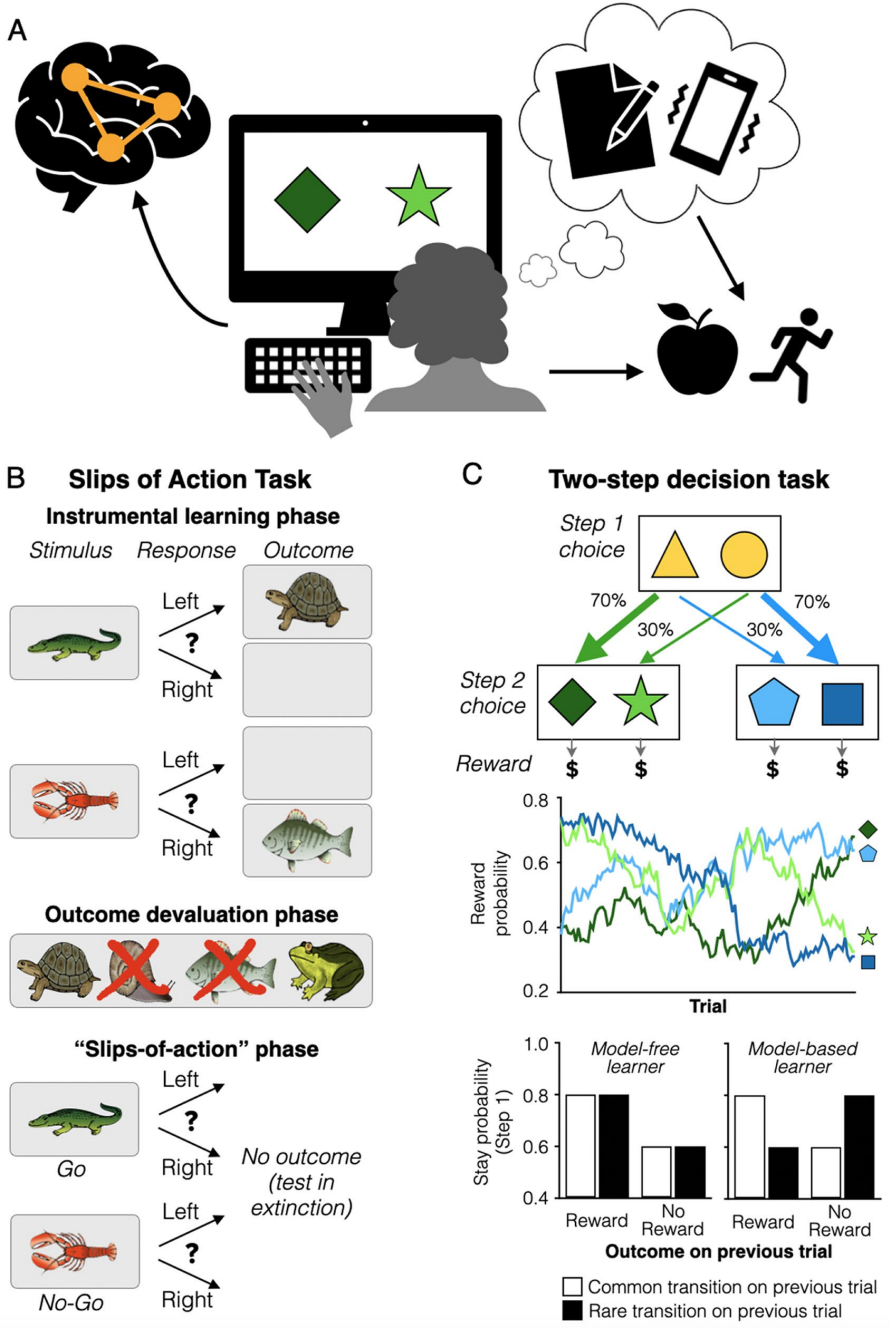
**(A)** Investigating habits. Understanding the role of habits in AN will likely require a multi-method approach. Development of rigorous task-based assessments is valuable to uncover cognitive mechanisms underlying habitual behavior. In addition, computerized tasks provide a way to more directly link behavior to dysfunction in neural circuits and regions through the use of neuroimaging. The addition of self-report questionnaires and ecological momentary assessments (daily diary methods) further support links between neurobiology and illness-related behaviors. **(B)** Slips of Action task. First, in the Instrumental Learning phase (top panel), participants learn which response in the presence of a discriminative stimulus leads to an outcome picture and reward (e.g., 5 points). Next, in the Outcome Devaluation phase (middle panel), participants are instructed that some outcomes no longer are valuable (lead to a loss of points/reward): devalued outcomes are “crossed out” while still valuable outcomes are shown not “crossed out”. In the “slips-of-action” phase (bottom panel), participants are shown the discriminative stimuli and must make appropriate responses to gain points and, to avoid losing points, not make responses that lead to devalued outcomes. In this phase, participants may respond automatically according to stimulus-response habits for stimuli that previously led to reward points, despite instruction that the outcomes no longer are valuable. Note that only a few example associations are depicted, but multiple discriminations usually are trained concurrently. **(C)** Two-step task. The transition structure between stages is designed to disambiguate “model-based” from “model-free” computational learning styles, believed to underlie goal-directed and habitual behaviors, respectively (top panel). In Step 1, participants choose between two cues that lead to Step 2, in which a new cue-pair is presented. Choice at Step 2 leads to reward (or punishment) with a probability that slowly changes across trials for each Step 2 cue, such that continuous learning is necessary (middle panel). Critically, each Step 1 cue is probabilistically associated with the transition to the Step 2 cue pairs—one choice leads to Step 2 cue pair 70% of the time but to the other cue pair 30% of the time. Each type of learning makes a different prediction about how a Step 1 choice is influenced by the final outcome at Step 2. A habitual approach is ignorant of transition structure and favors repeating Step 1 choices that ultimately result in reward, even by way of low probability transitions. For example, choosing the yellow triangle, then seeing the blue cues, choosing the dark blue square, then receiving reward, would lead to choosing the yellow triangle on the following trial. By contrast, a goal-directed approach uses transition contingencies to guide choices and the same experience should lead to switching to choose the yellow circle to get to the dark blue square and reward. Thus, model-based (goal-directed) learning predicts that the previous trial’s reward outcome will interact with whether it follows a Common (high probability) or Rare (low probability) transition to determine subsequent Step 1 choices, whereas model-free (habitual) learning predicts a main effect of reward (bottom panel)

The limited number of studies directly examining habits may stem from the inherent challenges of studying them in a laboratory setting [29] paired with additional challenges in eating disorder populations. In real-world contexts, habits develop gradually over time, and much of our understanding of habits comes from animal studies with extensive training sessions. One paradigm that captured habitual behavior over extended training relied on consumption of desirable foods until satiation [32] making it unsuitable for use in individuals with AN in addition to the practical difficulty with long training protocols. However, other approaches developed to study habits in humans (Fig. 3) have been employed widely in psychiatric illnesses (e.g., OCD [82]; substance use disorder [83]; alcohol dependence [84]) and have now been applied to the study of AN.

#### Outcome Devaluation Tasks

Outcome devaluation, decreasing the motivational value of an outcome (e.g., pairing a food with illness or feeding until satiation), is a standard tool to test whether behavior is habitual or goal-directed [84, 85, 86]. If an action is triggered by cues in the environment rather than the value of its associated outcome, then behavior should be insensitive to the value shift resulting from devaluation.

Godier and colleagues, who conducted one of the first task-based assessment of habits in AN, used two tasks with outcome devaluation procedures [88]. One, the Slips of Action task, pits habitual stimulus-response learning and goal-directed response-outcome learning against each other to assess their relative influence (see Fig. 3b). Participants learn whether to make a left or right button response in the presence of a discriminative stimulus to receive a specific outcome picture and reward. In an outcome devaluation manipulation, participants are informed which outcomes no longer are valuable and which still are valuable. Then, in a “slips-of-action” phase, participants are shown the discriminative stimuli and should respond to those that lead to still-valuable outcomes and not to those that lead to devalued outcomes. Stronger stimulus-response habits should yield more responses to the stimuli that previously led to reward despite the outcomes no longer being valuable. Across two experiments, the balance between habitual and goal-directed responding did not differ between individuals with AN and controls [88].

Another experiment reported by Godier and colleagues used an avoidance task in which participants learn by trial-and-error to make responses to avoid an aversive outcome (here an unpleasant noise, commonly a mild electric shock) signaled by discriminative cues. Following training, one outcome is “devalued”, in this study by disconnecting the headphone delivering noise to one ear, and participants continue to make responses to the discriminative cues. Habitual behavior is indicated by continued response to cues signaling devalued outcomes. In this study, individuals with AN and control participants did not differ significantly [88].

Two recent task-based assessments of habits among AN used Slips of Action tasks. Favier and colleagues found a shift towards habits among patients with the restricting subtype of AN [89], whereas Westwater and colleagues, who included only AN participants with the binge-eating/purging subtype, did not find significant differences between AN and control participants [62]. Thus, for outcome devaluation type tasks, a preponderance of the evidence does not indicate a stronger propensity towards habits among individuals with AN.

#### Two-Step Tasks

Two-step Markov decision tasks have been used extensively in psychiatric populations [83] and recently also with individuals with AN [90, 91]. The task design stems from the idea that habitual and goal-directed behaviors are associated with distinct computational algorithms identified as model-free and model-based learning [26, 38]. Whereas model-free learning is characterized by direct reinforcement of actions that lead to reward, model-based learning additionally accounts for task structure when evaluating actions. To capture this distinction, the task proceeds in two steps. Choices at the first step lead to distinct second steps at which choice can lead to reward (or punishment). Critically, choice at the first step is probabilistically associated with a transition to a given second-step cue pair. This transition structure allows dissociation of model-free and model-based learning: model-free behavior favors repeating first-step choices that result in reward at the second step, whether following a high- or low-probability transition, whereas model-based learning considers the transition probability when deciding the best first-step choice to get to the desired second-step outcome. Typically, reinforcement learning models are used to assess the model-based and model-free learning contributions for participants.

The first study using a two-step task to examine AN, administered versions with monetary and food outcomes [90] to examine illness-specific versus general habit tendencies and to address the possibility that monetary outcomes are less salient for inpatients (of relevance as attention and motivation may be critical for goal-directed learning on this task [92, 93]). Foerde and colleagues [90] found decreased goal-directed (model-based) learning among patients with AN while habitual (model-based) learning was unaffected—both for monetary and food outcomes and at the beginning and end of inpatient treatment (after treatment and weight restoration). Though the habitual component was not increased, the decrease in goal-directed control suggested a shift in the habit-goal balance towards habitual behavior. Indeed, this pattern has been found across other clinical populations [83, 94] and, in a large, online, non-clinical sample, reduced goal-directed control was associated with eating disorder-related symptomatology [95].

A recent study by Brown and colleagues [91] used two-step tasks with monetary gains or losses in adolescents with AN restricting subtype, AN binge eating/purging subtype, and control participants. For patients with the restricting subtype, model-based learning was significantly decreased for losses and at trend-level for gains, while model-free learning was decreased for losses but increased for gains. Patients with binge eating/purging subtype did not differ significantly from controls. The earlier study by Foerde et al. [90], though not designed to compare restricting and binge-eating/purging subtypes of AN, reported more pronounced decreases in model-based learning among the binge-eating/purging subtype [90].

Overall, studies with Two-step tasks point to a tendency for habitual control of behavior among individuals with AN, but the exact conditions under which this occurs, and for which subgroups, awaits clarification.

#### Pavlovian Instrumental Transfer

Pavlovian Instrumental Transfer (PIT) tasks, also inspired by animal experiments, have garnered increasing interest for studies in psychiatric populations and addiction [84, 96]. The PIT task comprises several phases: In one phase, associations are learned between responses and outcomes (Instrumental) and in a separate phase, associations are learned between cues and outcomes (Pavlovian). In a Transfer phase, the acquired instrumental responses are made, sometimes in the presence of the acquired Pavlovian cues. Of interest is whether responding for an outcome is increased in the presence of a Pavlovian cue not previously associated with the instrumental response. The PIT task can be paired with outcome devaluation and invigorated responding can happen for outcomes that are no longer valuable, suggesting habitual responding.

A study by Vogel and colleagues used a PIT task [97] in which participants responded on different keys to earn ‘chocolate’ and ‘vegetable’ coins in the instrumental phase. Participants learned to associate abstract stimuli with chocolate and vegetable images in the Pavlovian phase—learning of associations was assessed by asking about expectancy of a chocolate or vegetable images on each trial. In the transfer phase, participants continued to make responses to earn ‘chocolate’ and ‘vegetable’ coins, on a subset of trials in the presence of the Pavlovian cues. While no overall differences were seen between patients and controls, there was an association between responding for low-calorie (vegetable) outcomes and eating disorder pathology, suggestive of more rigorous responding for outcomes associated with restrictive eating.

Though not the focus of the Vogel study, several aspects point to considerations for future studies. Individuals with AN exhibited poorer learning of the Pavlovian associations than did controls. Moreover, fewer than one third of the AN group were classified as “aware” of those associations (compared with more than half of the control group), based on whether expectancy ratings during the Pavlovian phase differed significantly between cues at the end of training. This distinction was critical because PIT effects only were observed among “aware“ individuals. Consequently, despite an initially adequate sample, the sample in which the effect could be studied was quite small. Deficits in learning have been reported among individuals with AN in previous studies [95, 96, 97] and differential acquisition of associations could, as suggested by the authors of the PIT study [97], be addressed with more extensive training to ensure that groups are equated on relevant factors.

### What Can be Learned to Make the Most of Future Research on Habits?

Though only a handful of experimental examinations of habits among individuals with AN exist, most are recent, emerging following initial calls for investigations of the role of habit in AN [12, 21]. While an overall summary of the reviewed studies appears inconclusive, with group differences emerging in about half, the pattern of results suggests some directions for future work. Among the studies using tasks with outcome devaluation procedures [62, 88, 89], only one found group differences [89], whereas both studies using Two-step tasks found differences. Some limitations in the study using the PIT paradigm preclude firm conclusions. The pattern of results could suggest that tasks vary in their sensitivity or suitability when examining individuals with AN. Additionally, available studies included populations that differed across multiple dimensions. Task and population factors to consider in future studies are outlined below.

#### Task Factors

##### Paradigms

The Two-step tasks appeared most likely to identify group differences [90, 91] (but also included the largest sample sizes, about double that of studies using the outcome devaluation tasks). The Two-step and outcome devaluation tasks differ in how habitual and goal-directed behaviors are assessed. The outcome devaluation procedures are explicit, directly instructing participants as to which outcomes are valuable and should be responded to. By contrast, the Two-step task relies on observing behavioral patterns as a function of underlying task structure, a design difference that may render this class of tasks more sensitive. Another possibility is that computational model-based data analyses allow for more sensitive assessment of the dynamics underlying behavior. Nonetheless, measures of goal-directed control in the two types of tasks are correlated across individuals [101].

The PIT task, despite unclear results regarding AN, could be an interesting paradigm for future studies. It may be relevant when cue-triggered relapse is a concern and has increasingly been used to examine smoking and alcohol use [102]. Numerous variations on the paradigm exist [84, 96] and it could provide a promising complement to the Slips of Action and Two-step tasks that dominate current research.

##### Valence

Appetitive (gaining rewards) vs. aversive (avoiding losses) contexts is a theoretically relevant factor when considering learning in AN [103]. Two studies included aversive outcomes, with one finding no group differences [88] and one finding decreased habitual and goal-directed behavior with punishment [91]. Valence does not account for the current pattern of results across studies, but examination of avoidance habits is of clear importance when considering restrictive eating behavior in AN. Notably, avoidance habits may be more complex than, and not just mirror reflections of, approach habits [104], presenting additional challenges.

##### Domain Specificity

Inconsistent findings across studies could arise if habit tendencies mainly emerge for illness-related behaviors and only sporadically as a general tendency. One study incorporated illness specific outcomes (food) in comparison with monetary outcomes and found group differences regardless of outcome type, indicating a domain-general effect [90]. Consistent with this, the EMA study of daily habits found that both food and hygiene habits were more frequent among individuals with AN than control participants [81]. Thus, available evidence points towards general effects, if a predilection towards habits is present in AN.

#### Population Factors

##### Subtype

Inclusion of individuals with restricting or binge eating/purging subtype of AN varied across studies: some included both subtypes [88, 90, 97], some focused either on restricting [88, 89] or binge-eating/purging [62] subtype, and only one study was designed to compare subtypes [91]. However, differences relative to control participants were found both for the restricting [89, 91] and the binge-eating/purging subtype ( [90]; post hoc, as the study was not designed to study subtypes). Although individuals diagnosed with different subtypes may not differ on central illness behaviors (e.g., restrictive eating [105]), they may differ in learning-related disturbances, with consequences for how behavior is acquired and changed. Clarifying such potential differences could be valuable for understating the role of habits.

##### Treatment Stage, Medication, and Comorbidity

Across studies, patients may have differed as to when in treatment assessment occurred (if in treatment), with implications for how underweight patients were at the time of study, though it should be noted that the study by Foerde et al. [90] found deficient goal-directed behavior both at the beginning (within 1 week of admission) and end of treatment and weight restoration. Inclusion/exclusion criteria also varied regarding medications and co-morbid diagnoses, potentially relevant for habitual and goal-directed behavior (e.g., OCD). Careful consideration and reporting of these factors will facilitate interpretation of future studies.

##### Age

The reviewed studies included mostly adults [62, 88, 89], with some also including older adolescents [90] or a range from adolescent through young adults [97], and one study including only adolescents [91]. Though results did not appear to vary by age, further studies of the development of maladaptive behaviors among adolescents will be important. Adolescence is a unique time of rapid brain development while learning and exploring new behaviors [106]. Eating disorders often emerge during adolescence [107], and the incidence of AN may be increasing among younger adolescents [108]. Given the potential for long-term illness, understanding mechanisms, such as habits, that may contribute to persistence is critical.

## Conclusions

Examination of the role of habits in AN remains nascent and more studies are needed. Pursuit of two distinct paths in parallel may offer progress.

One path must grapple with the challenges of experimental examination of habits in general. Habits are difficult to capture in the lab and available paradigms are imperfect [29]. Laboratory tasks adapted from animal models often involve learning simple contingencies and are quickly acquired by humans. While this approach improves feasibility, it raises questions about whether these tasks adequately capture the complexity and gradual formation of real-world habits [109, 110]. This calls for continued development of paradigms and incorporation of promising paradigms used trans-diagnostically, such as PIT tasks [102]. Additionally, approaches better suited to capture habits as they occur outside the lab, in the real world (e.g., EMA; [81]) should be adopted. This could also ensure that habitual behaviors relevant for illness are understood, which is needed for effectively targeted treatment. Overall, this path favors innovation and implementation of novel approaches.

A second path favors coalescing around shared paradigms across researchers to better understand the role of varied characteristics across participants: What is the role of development? Is habit proneness vs. resistance characteristic of (pre)adolescence, when illness commonly starts? What is the role of starvation in acquiring and maintaining habits? Are there individual differences that could inform illness course? Studies are often conducted across age groups, illness stages, and severity. Variations in choice of paradigms, or analysis streams of similar paradigms, make comparisons difficult. Standardization would facilitate comparison across studies to elucidate for whom habit may play a role in illness. Open sharing of tasks and resources would be a simple way to move towards standard procedures.

Hopefully these potentially promising avenues will be pursued. The role of habits in AN deserves continued, rigorous study given the need to understand the neurocognitive mechanisms of AN.

## Key References

- Seidel, M., et al., *Increased Habit Frequency in the Daily Lives of Patients with Acute Anorexia Nervosa.* Nutrients, 2022. 14(19): p. 3905.
-
  - This study is the first to examine habits among individuals with anorexia nervosa using ecological momentary assessment and found increased habitual behaviors not only related to eating but also in broader contexts like hygiene.
- Favier, M., et al., *Cholinergic dysfunction in the dorsal striatum promotes habit formation and maladaptive eating.* Journal of Clinical Investigation, 2020. 10(15): p. 111 − 16.
-
  - This study reported enhanced habit formation among individuals with anorexia nervosa restricting subtype. Additionally, it was demonstrated in mice that cholinergic dysfunction in the dorsal striatum facilitated a shift from goal-directed to habitual behavior, providing a mechanistic link between neural dysfunction and maladaptive eating.
- Brown, C.S., et al., *Greater reliance on model-free learning in adolescent anorexia nervosa: An examination of dual-system reinforcement learning.* medRxiv, 2024.
-
  - This study examined habitual and goal-directed behavior using a Two-step task with both monetary reward and loss conditions among adolescents with either binge-eating/purging or restricting subtype of anorexia nervosa. Among individuals with restricting subtype, a general tendency for decreased goal directed behavior was found, whereas habitual behavior increased with reward outcomes and decreased with loss outcomes.

## Data Availability

No datasets were generated or analysed during the current study.

## Footnotes

1 The Creature of Habit Scale may have limitations for use in eating disorders research. The Automaticity subscale consists mainly (all but one) of questions related to impulsivitiy around food and eating rather than food restriction [62].
